# *‘We spray and walk away’*: wall modifications decrease the impact of indoor residual spray campaigns through reductions in post-spray coverage

**DOI:** 10.1186/s12936-020-3102-6

**Published:** 2020-01-17

**Authors:** Mercy A. Opiyo, Krijn P. Paaijmans

**Affiliations:** 1 ISGlobal, Hospital Clinic, University of Barcelona, Barcelona, Spain; 2 Centro de Investigação em Saúde de Manhiça (CISM), Maputo, Mozambique; 3 Center for Evolution and Medicine, School of Life Sciences, Arizona State University, Tempe, USA; 4 The Biodesign Center for Immunotherapy, Vaccines, and Virotherapy, Arizona State University, Tempe, AZ USA

**Keywords:** Insecticide, Wall modification, Residual efficacy, Communities, Vector control, Compliance, Elimination

## Abstract

Malaria prevalence has significantly reduced since 2000, largely due to the scale-up of vector control interventions, mainly indoor residual spraying (IRS) and long-lasting insecticide-treated nets (LLINs). Given their success, these tools remain the frontline interventions in the fight against malaria. Their effectiveness relies on three key ingredients: the intervention, the mosquito vector and the end-user. Regarding the intervention, factors such as the insecticide active ingredient(s) used and the durability and/or bio-efficacy of the tool over time are critical. For the vectors, these factors include biting and resting behaviours and the susceptibility to insecticides. Finally, the end-users need to accept and properly use the intervention. Whilst human attitude and behaviour towards LLINs are well-documented both during and after distribution, only initial coverage is monitored for IRS and in a few geographic settings the residual efficacy of the used product. Here, the historical evidence on end-users modifying their wall surfaces post-spraying is presented, a behaviour that has the potential to reduce actual IRS coverage, effectiveness and impact, as fewer people are truly protected. Therefore, clear guidelines on how to monitor IRS acceptability and/or coverage, both before, during and after spraying, are urgently needed as part of the Monitoring and Evaluation of malaria programmes.

## Background

Insecticide-treated nets (ITNs), later replaced by long-lasting insecticidal nets (LLINs), and indoor residual spraying (IRS) are core vector control interventions in malaria-endemic settings and have averted 663 million clinical malaria cases (three-quarters of the gains achieved) between 2000 and 2015 [1].

Albeit the huge success in the fight against malaria, malaria cases are on the rise again since 2016 [2, 3] which endangers the long-term goals of the Global Technical Strategy (GTS) for Malaria [4]. As LLINs and IRS will remain the backbone of malaria control and elimination efforts, optimized approaches to improve these interventions are required to further reduce the burden.

In addition, current vector control tools face several challenges, which include sub-optimal user compliance [5] the rapid spread of insecticide resistance [6, 7] mosquito behavioural changes after implementation of interventions [8], and implementation costs. All these factors combined greatly undermine the effectiveness of current intervention packages [2]. Whilst many studies focus on the vector (resistance, time and place of biting), the critical component of how human behaviour impacts the efficacy of vector control tools has been largely overlooked for IRS. In this article, after illustrating the importance of IRS in malaria control and discussing the current indicators that are used to evaluate its impact, the focus will shift to a critical indicator that is currently not measured: Changes made to insecticide-treated walls (wall modifications) by household owners over time following IRS implementation.

### IRS and the fight against malaria: past, present, and future

Indoor residual spraying (IRS) has been a key intervention in the fight against malaria mosquitoes since the discovery in 1939 that chemicals like DDT had insecticidal properties and could aid public health by reducing the vector population size [9]. To reduce the high malaria burden in the 1960s, the World Health Organization (WHO) introduced large scale use of IRS to target indoor resting mosquitoes as the primary vector control method [9]. To supplement this effort, methods such as larval control and building mosquito-proofed houses [10] were encouraged too. However, IRS was later reduced to fewer countries due to challenges such as insecticide resistance due to over-reliance on one class of insecticide (the organochloride DDT), logistical constraints, sustainability, economic and financial issues [10].

However, in 2006, the WHO recommended IRS again as a tool to reduce or interrupt local malaria transmission in different epidemiological settings. As a result, several countries introduced and/or expanded their IRS programmes [11], with dedicated funding from President’s Malaria Initiative (PMI) and the Global Fund to Fight AIDS, Tuberculosis and Malaria (GFATM). IRS is believed to have contributed to about 14% and 16% of overall malaria cases prevented and reduction in malaria prevalence, respectively, between 2000 and 2015 in the WHO Africa region [1]. As IRS remains an integral part of vector control efforts, partly because of the widespread resistance to pyrethroids used in LLINs [12, 13], huge investments are channeled to it [3, 14].

### How do we currently monitor IRS efficacy?

The effectiveness of LLINs and IRS relies on (1) the biology and behaviour of mosquito vectors (their biting and resting behaviour as well as their susceptibility status to insecticides), (2) the insecticide(s) selected (active ingredient, bio-efficacy (in case of LLINs) and residual efficacy (IRS) over time), durability (LLINs), and (3) the acceptability and use of the interventions as well as their coverage [15–18]. The latter, coverage, is normally estimated by calculating the proportion of the households that own 1 LLIN per 2 persons, or the proportion of houses or structures or households that have been sprayed in the case of IRS [19–22]. A certain coverage threshold (believed to be 60% for LLINs [17] and 80% for IRS [19, 22] is envisioned for the tools to work optimally as they work at a community level: the greater the coverage, the higher the impact [18, 22, 23].

For LLINs, the most widely used vector control intervention, there are clear guidelines on how to monitor relevant indicators to assess their protective efficacy post-distribution [21, 22, 24]. These indicators, which include coverage, usage, net maintenance and killing efficacy, are routinely monitored at an annual interval after net distribution [25–27]. However, for IRS, apart from assessing the initial coverage (at time of application), only the quality of the IRS product may be assessed post-spraying through standard residual efficacy tests using WHO cone bioassays [19, 28]. This is, however, not standard practice in all malaria-endemic countries, and if implemented, often only carried out in a handful of houses per province or even country, and halted when mosquito mortality falls below 80%.

LLIN studies show that community acceptance and uptake is key to the success of vector control. For IRS, advocacy campaigns before the implementation of IRS are geared towards preparing communities for IRS to achieve high initial coverage. However, after a house/structure has been sprayed, there is no follow-up on whether the treated surfaces remain covered until the next spray cycle (or at least up to the point where the insecticide lost its residual efficacy). The WHO and other institutions have not yet set forth guidelines to aid national malaria control programmes (NMCPs) and partners on how to monitor post-spray owner compliance. Current guidelines to assess the effectiveness of IRS programmes include initial coverage (number of structures covered) and the residual efficacy of the product sprayed [19] (Table 1). For simplicity, here it is assumed that adequate entomological surveillance is in place, to ensure that targeted vector populations do rest indoors, and are susceptible to the insecticides selected (although this is unlikely always the case).

**Table 1:** Currently recommended indicators for monitoring the effectiveness of IRS programmes

Program	IRS indicator	Reference
World Health Organization (WHO)	Coverage: ‘the proportion of structures/houses sprayed in relation to those not sprayed (proportion of structures/houses sprayed in relation to those targeted for spraying)’. This is done at the district, region, country, province, and global scale Residual efficacy: ‘the quality of IRS, impact, insecticide dosage and longevity on treated surfaces is routinely measured by WHO cone bioassay using susceptible strains maintained at a central laboratory’ Social performance: ‘the perception of the community towards IRS can be assessed through community knowledge, attitude, behavior and practice (KABP) surveys’. Such surveys are not required to run an IRS program but are reserved for situations where community-related IRS problem may arise	[48]
Roll Back Malaria (RBM)	Population coverage: ‘Proportion of households sprayed by IRS in the last 12 months’	[49]
UNICEF	Households covered by vector control: ‘Proportion of households sprayed by IRS in the last 12 months’. Universal coverage of vector control: ‘Proportion of households sprayed by IRS within the last 12 months’	[50]

### IRS and society: the human attitude and behaviour towards IRS

**Fig. 1:**
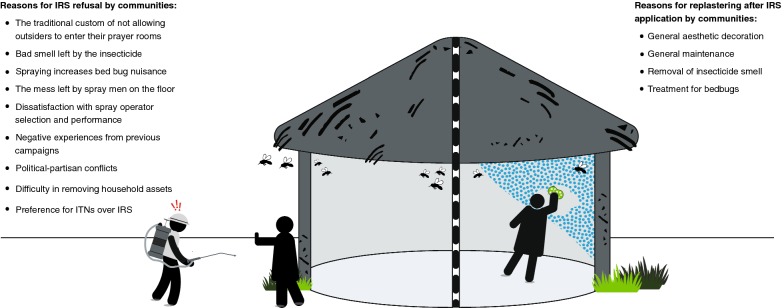
Some reasons for IRS refusal (on the left) and for wall modifications post-application (on the right) by communities [32, 33]

One of the WHO-recommended, but not required, indicators for effective implementation of IRS is ‘social performance’. Social studies that exist assess the acceptability of IRS prior and/or during the implementation phase, to understand why IRS is refused and to evaluate if action can be taken to mitigate this (i.e. through improved community awareness) [29–32]. As alluded to before, LLIN indicators such as usage, coverage, and durability are monitored annually post-distribution using existing guidelines [20, 21]. But when it comes to IRS, once spraying is done, only monitoring of the product’s residual efficacy occurs sporadically via wall bio-assays. The extent to which insecticide-treated wall surfaces are modified post-application, and why, are currently not being monitored, yet home-owners are known to wash and/or brush, (re)plaster and/or (re)paint their walls over time. These activities occur for a variety of reasons, such as the dislike of the smell or coloration of walls after IRS application, mistrusting the government/implementers [33, 34] or due to non-IRS related factors such as scheduled maintenance/renovations and religious/cultural practices [35] (Fig. 1). As this particular indicator (coverage over time post-spraying) is not routinely monitored by NMCPs programmes and/or partners, it is impossible to understand its significance and its impact.

**Fig. 2:**
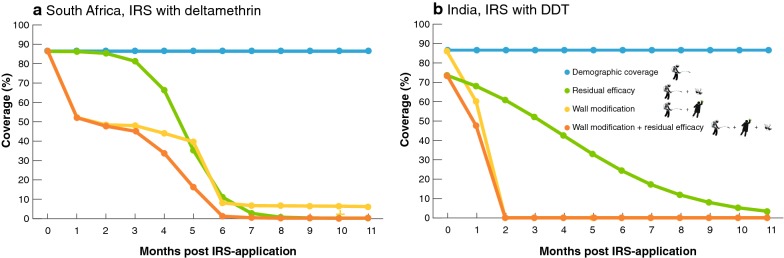
The effect of wall modifications and waning residual efficacy on the actual coverage of IRS campaigns. **a** Data from mud houses in South Africa (IRS with deltamethrin in 1999) and **b** from Koraput district in India (IRS with DDT 2004). The blue line represents actual (demographic or initial coverage as reported by programs and/or partners determined at the time of spraying), the yellow line represents observed wall modifications following real IRS-applications, the green line represents the residual bioefficacy on non-modified walls as monitored through WHO cone bioassays and the orange line represents the predicted impact of wall modifications combined with a waning residual efficacy on the effective IRS coverage. Logistic binomial models were fitted through actual residual bioefficacy data, obtained from (deltamethrin) [15] and (DDT) [43]. Bayesian models were fitted using Hamiltonian Monte Carlo sampling methods [51, 52]. Four chains were initialised to assess the convergence of 1000 iterations, the first 500 of each were discarded as burn in. The posterior distributions of parameters (4000 iterations) and 90% Bayesian credible intervals were estimated, posterior checks were performed using shinystan (version 1.0.0) [53] and visually confirmed to fit the data. The caveats for the South Africa data are: (1) repeated measures, as walls were washed or replastered on more than one occasion during the year, which leads to an overrepresentation of the extent of wall modifications, (2) initial coverage not being reported, necessitating us to use the self-reported 86.6% for IRS during the past 2 years, (3) no information on start date of IRS, so we assumed month 1 to be November, matching text with table. The caveat for the data from India includes initial coverage (reported for households and structures, we opted to show household-level data, as modification data were also reported at that level). For both studies it is not clear (probably not assessed) if all rooms and all walls in each room had been modified, or if only part of the house/structure was affected. Note that additional data (South Africa, concrete houses; India, Malkangiri district) are shown in Additional file 1: Fig. S1, and show similar patterns

However, two previous studies have shown that IRS coverage can decrease rapidly over time as a result of wall modifications post-spraying. Figure 2 shows data from South Africa (IRS with deltamethrin in 1999) and India (IRS with DDT in 2004). In both studies, IRS coverage (here defined as walls left untouched after IRS application) rapidly declined post-application. In South Africa, coverage decreased to 50% within 3 months after spraying [36] (Fig. 2a), and in India, 80% of the houses were replastered within two to 3 months following the IRS campaign (Fig. 2b) [33]. By month 3 or 4 (depending on the district) walls in all the sprayed houses had been replastered [33] (Fig. 2b). Although there may be some caveats with the study designs (see the legend of Fig. 2), the figures clearly illustrate there may be a serious challenge with IRS if communities today behave in similar ways.

### What is the true impact of the IRS?

To date, the studies from India and South Africa provide the only longitudinal data on wall modifications post-IRS. Whilst it is not known how representative the data from India and South Africa are to other settings in Africa where IRS has been or is being implemented, wall modifications could reduce or potentially completely remove the bioavailability of insecticides. This may change the outcomes of IRS impact evaluations, as this indicator is not included in models that estimate the efficacy (i.e. number of structures/houses sprayed and/or the number of people protected) of IRS campaigns [14, 37–39]. These estimates are mainly based on initial coverage, the vector susceptibility status to insecticides, and the residual efficacy of the product used [23, 38, 40]. In addition, studies that aim to evaluate the additional benefits of IRS combined with LLINs have concluded that no or little incremental benefits exist when the two interventions are combined [41, 42]. These latter studies have, however, only considered initial coverage and could have underestimated the additional protective value of two interventions combined, if study communities modified their walls post-IRS application.

Apart from changes in coverage post-spraying, there is little known about the impact of wall modifications on the residual efficacy of the insecticide. This will likely depend on a variety of factors, including the type and extent of the modification, as well as the materials used to prepare and modify the walls. Replastered walls have been shown to still kill malaria vectors, although mortality was significantly lower on replastered walls [33]. Having said that, looking at the patterns in Fig. 2, and considering the residual efficacy of deltamethrin (which ranges from 1 to 3 months) [15] and DDT (2 months) [43] it is fair to assume that the observed wall modification behaviours in South Africa and India will have impacted control efforts. To illustrate this, existing data on wall modifications and residual efficacy are combined in a single plot (Fig. 2), assuming that waning residual efficacy equals loss in coverage (i.e. 70% mortality in cone bio-assays translates to 70% of the houses killing all mosquitoes, while 30% kill none). The situation is more complex, as different houses have different residual efficacies and attract different numbers of different vector species, but Fig. 2 illustrates how wall modification could severely impact IRS efficacy, and hence the actual number of people protected.

Although deltamethrin (and other pyrethroids) and DDT are now less frequently used in IRS campaigns due to resistance and/or health concerns [12, 28, 44–46] Actellic^®^300CS (Syngenta, Switzerland), SumiShield^®^50WG (Sumitomo, Japan) [14, 47], both with residual efficacies of > 6 months, will not be as impactful as currently assumed if household members modify their walls post-spraying.

## Conclusions

To understand the true impact of IRS campaigns, IRS coverage post-implementation needs to be evaluated as part of Monitoring and Evaluation activities, to estimate the number of people protected. By creating guidelines for malaria control programmes and partners, the extent of wall modifications following a successful IRS campaign can be monitored and appropriate actions taken to either prevent or mitigate unwanted human behaviours. These include actions like improved community engagement and education, and/or re-spraying of modified walls. This will ensure that IRS campaigns achieve optimal protection, thereby reducing malaria morbidity and mortality even further.

## Supplementary information

**Additional file 1: Figure S1.** The effect of wall modifications and waning residual efficacy on the actual coverage of IRS campaigns.

## Data Availability

Data shown in this article (Fig. 2) are obtained from the scientific literature and all citations can be found in the bibliography.

## Abbreviations

LLINs: long-lasting insecticidal nets
ITNs: insecticide-treated nets
IRS: indoor residual spraying
GTS: Global Technical Strategy
DDT: Dichlorodiphenyltrichloroethane
WHO: World Health Organization
GMEP: Global Malaria Eradication Program
PMI: President Malaria Initiative
NgenIRS: next-generation indoor residual spraying
GFATM: Global Fund to Fight AIDS, Tuberculosis and Malaria
PMI AIRS: President Malaria Initiative Africa Indoor Residual Spraying
RBM: Roll Back Malaria
UNICEF: United Nations International Children’s Emergency Fund
CISM: Centro de Investigação de Saúde de Manhiça
