# Supplementary figures and images for: ‘We spray and walk away’: wall modifications decrease the impact of indoor residual spray campaigns through reductions in post-spray coverage
Source: Malar J. 2020 Jan 17;19:30. doi: 10.1186/s12936-020-3102-6 (PMC6969461; doi:10.1186/s12936-020-3102-6)

### A) South Africa, IRS with deltamethrin

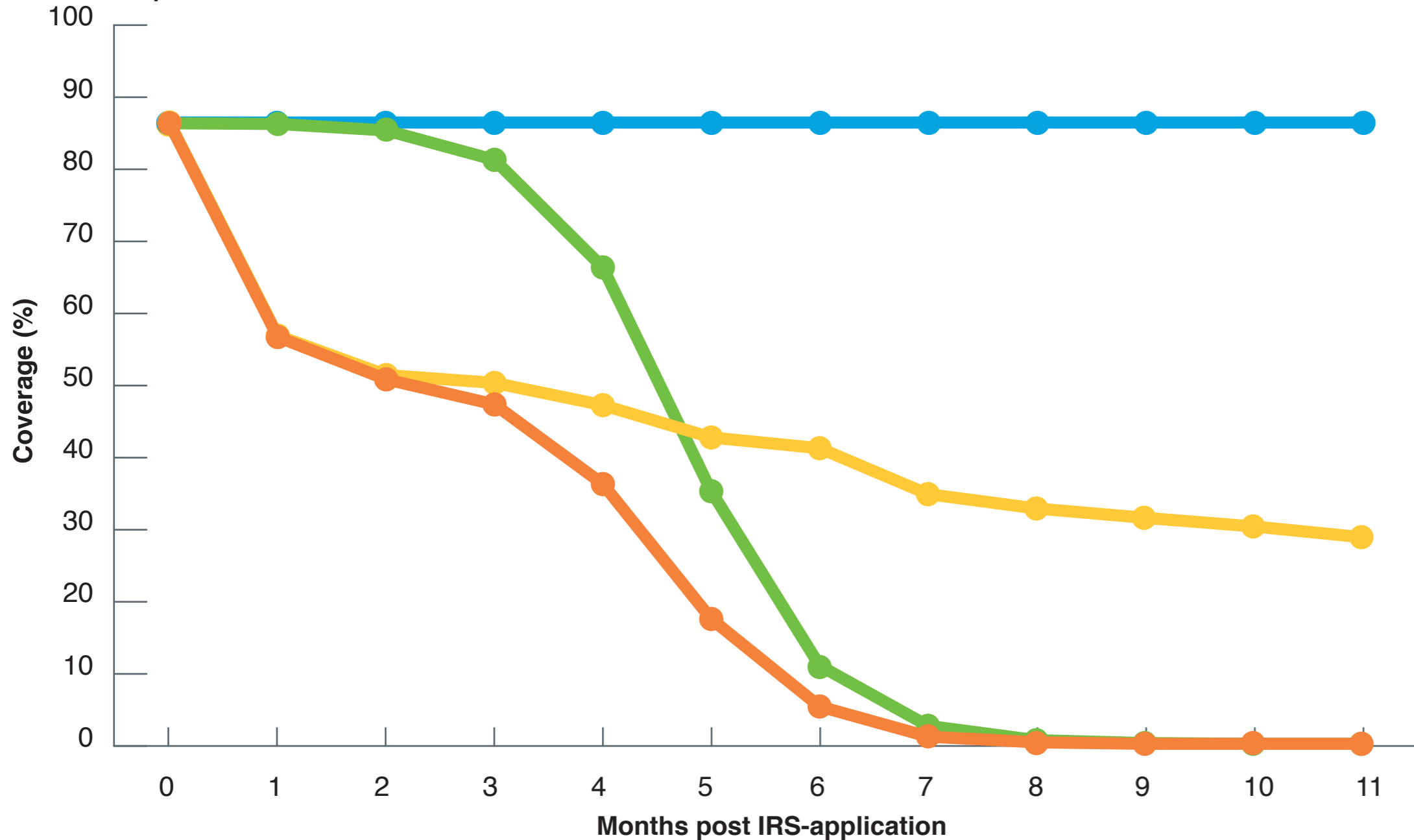

### B) India, IRS with DDT

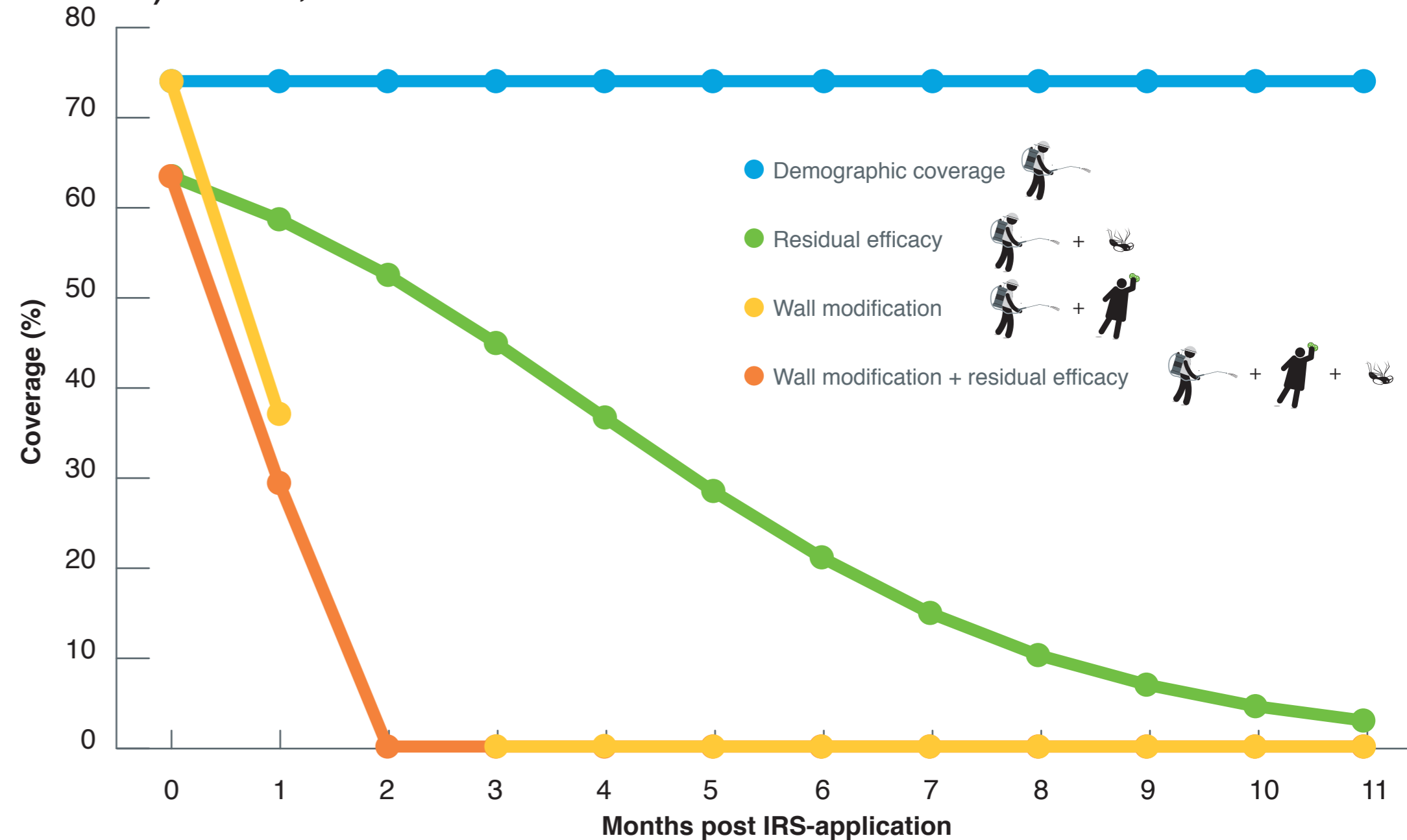

Supplement: Supplementary file 1 — Additional file 1: Figure S1. The effect of wall modifications and waning residual efficacy on the actual coverage of IRS campaigns. [file 12936_2020_3102_MOESM1_ESM.pdf]
